# Risk factors and causes of early death in germ cell tumors: a Global Society for Rare GU tumors study

**DOI:** 10.1038/s41416-026-03484-0

**Published:** 2026-06-02

**Authors:** Michal Mego, Edgar Israelyan, Robert J. Hamilton, Axel Heidenreich, Umberto Basso, Patrizia Giannatempo, Tomas Buchler, Klaus-Peter Dieckmann, Robert Huddart, Bruno Vincenzi, Kwonoh Park, Jorge Aparicio, Alexei Tryakin, Ali Amiri, Pavlina Malcharkova, Melanie Claps, Marija Miletic, Lucia Chaloupkova, Markus Angerer, Philippe E. Spiess, Ugo De Giorgi

**Affiliations:** 1 Faculty of Medicine, Comenius University and National Cancer Institute, Bratislava, Slovakia; 2 N.N. Blokhin National Medical Research Center of Oncology, Moscow, Russia; 3 Princess Margaret Cancer Centre, Toronto, ON Canada; 4 Department of Urology, Uro-Oncology, Robot Assisted and Specialized Urologic Surgery, University Hospital Cologne, Köln, Germany; 5 Oncology Unit 1, Department of Oncology, Istituto Oncologico Veneto IOV - IRCCS, Padova, Italy; 6 Genitourinary Oncology Unit, Department of Medical Oncology and Ematology, Fondazione IRCCS Istituto Nazionale dei Tumori, Milan, Italy; 7 Motol University Hospital, Prague, Czech Republic; 8 Asklepios Klinik Altona, Dept, Urology, Hamburg, Germany; 9 The Royal Marsden NHS Foundation Trust, London, UK; 10 University Campus Bio-Medico, Rome, Italy; 11 Hanyang University Seoul Hospital, Hanyang University College of Medicine, Seoul, Korea; 12 University and Polytechnic Hospital La Fe, Valencia, Spain; 13 Department of GU Oncology, Moffitt Cancer Center, Tampa, USA; 14 Department of Experimetal Medicine, University of Salento, Fazzi Hospital, Lecce, Italy

**Keywords:** Germ cell tumours, Chemotherapy

## Abstract

**Background:**

Germ cell tumors (GCTs) are highly curable, yet a subset of patients with metastatic disease experience early death (ED) soon after starting first-line chemotherapy. These patients are underrepresented in trials, and risk factors remain unclear.

**Methods:**

We performed a retrospective multicenter cohort study including adults ( ≥ 18 years) with metastatic GCT who died within 3 months of completing their last cycle of first-line chemotherapy. Primary outcomes were cause and timing of death; secondary endpoints included clinical predictors of acute respiratory failure (ARF) and very early death ( ≤ 30 days).

**Results:**

Among 102 patients (1.7% of treated cases), 69.6% had non-seminoma, 83.3% testicular primaries, and 67.6% poor-risk disease. Median time to death was 28 days (range, 2–179). Leading causes were ARF (34.1%), disease progression (16.7%), septic shock (15.7%), hemorrhage (12.7%), and cardiovascular events (4.0%). ARF correlated with > 50% lung involvement, dyspnoea, and haemoptysis, but not choriocarcinoma histology or bleomycin use. Mostly, ED (51%) was associated with liver metastases, massive lung involvement, β-hCG > 50,000 mIU/mL, ECOG 2–3, elevated neutrophil/lymphocyte ratio, and need for intensive care (all *P* < 0.05).

**Conclusions:**

Early death in metastatic GCT, though rare, remains a critical clinical issue. Early identification, adapted induction regimens, and optimized supportive care may help prevent avoidable mortality.

## Introduction

Testicular germ cell tumors (GCTs) are the most common malignancies in young men aged 20–40 and serve as a model for curable cancer [1, 2]. Over 80% of patients with metastatic testicular GCT achieve long-term remission with cisplatin-based chemotherapy, though cure rates vary between 50% and 98% depending on the prognostic group [3, 4].

Despite these favourable outcomes, a small subset of patients—particularly those with advanced disease—experience early death (ED), typically within the first month after initiating systemic therapy [5]. Reported early mortality during first-line chemotherapy ranges from 0.8% to 2.2% but can exceed 50% in patients with poor performance status and extensive disease [5–8]. Reportedly, the highest risk is seen in those with poor prognosis features, including massive lung metastases and markedly elevated β-human chorionic gonadotropin (β-hCG) — a presentation known as “choriocarcinoma syndrome”[9]. The likely cause is acute respiratory failure from intra-alveolar haemorrhage and tumor necrosis, compounded by infection and chemotherapy-induced neutropenia [10, 11].

However, EDs in GCTs are not limited to this syndrome. Fatal events such as pulmonary embolism, myocardial infarction (MI), septic shock, and intratumoral haemorrhage can also occur, independent of histology [8, 12–14]. Such cases underscore the limitations of current IGCCCG risk stratification and the need for better identification of “super-high-risk” patients. ED patients are often excluded from clinical trials. Accordingly, their characteristics remain underreported, and importantly, the IGCCCG classification update did not systematically document EDs [4]. Only few retrospective studies and case reports have highlighted the urgent need to define this super-high-risk subgroup [6, 7, 15–17].

Previous studies suggested that “super-high-risk” patients with GCTs are those who, in addition to the IGCCCG poor-risk classification, present with additional adverse features such as older age, liver metastases, lung involvement exceeding 50%, and markedly elevated β-hCG and/or lactate dehydrogenase (LDH) levels [6, 7, 11, 16]. A distinct subgroup includes patients with the choriocarcinoma-syndrome phenotype, characterized by baseline dyspnoea, diffuse pulmonary metastases and β-hCG > 50,000 IU/L, who are at risk of fatal acute respiratory failure (ARF) within 7–10 days of initiating bleomycin-etoposide-cisplatin (BEP) chemotherapy [9, 11, 18]. Another super-high-risk profile includes patients with bulky mediastinal or extragonadal non-seminomatous GCTs with vascular invasion, which predisposes them to intralesional haemorrhage and tumor-lysis-associated cytokine storms [7, 10].

Current knowledge on early mortality in GCT patients is limited by several key gaps. Prospective data on ultra-early toxic deaths are lacking, as most clinical trials exclude patients who die before the first restaging. Additionally, there is no standardized definition of ED, with existing studies variably using 14-, 30-, or 42-day timeframes—hindering comparability and meta-analysis. Finally, while induction regimens such as Baby-BOP (cisplatin, vincristine, and bleomycin) or CBOP (carboplatin/bleomycin/vincristin/cisplatin) and/or initial dose reduction show promise, they are supported only by single-institution data, highlighting the need for randomized, multicentre validation [16, 19, 20].

This study aims to characterize GCT patients who die within 3 months of completing their last cycle of first-line chemotherapy. By identifying tumor and patient features associated with ED, as well as underlying mechanisms, we hope to inform risk stratification and guide tailored interventions for this vulnerable group.

## Patients and methods

### Patients

This was a retrospective cohort study including male patients, at least 18 years old, diagnosed with GCTs between the years 2000 and 2024 (Supplementary Fig. 1). The included patients had died within three months of completing their last cycle of first-line chemotherapy (or earlier) and had received systemic anticancer therapy, defined as having at least the first dose administered or a clear intention to treat. This project utilized an Excel database that contained data related to patient and tumor characteristics, treatments, and outcomes from participating centers. The submission for IRB approval was processed by each participating investigator at their local IRB committee. Each participating center or investigator had appropriate access to the database.

### Pretreatment evaluation

In cases with testicular primary tumors, orchiectomy was performed, and histological confirmation was obtained based on the World Health Organization (WHO) classification [21]. In rare cases, diagnosis was made without biopsy, relying on clinical presentation and markedly elevated serum alpha-fetoprotein (AFP) and/or β-hCG levels.

Baseline assessments included full physical examination and laboratory testing: complete blood count, serum electrolytes, renal and liver function tests, LDH, β-hCG, and AFP levels. Imaging included chest radiography, abdominal ultrasound, and CT scans of the chest, abdomen, and brain. Tumor staging, including serum tumor marker (S) staging, was performed according to the American Joint Committee on Cancer (AJCC) TNM classification system for testicular GCTs (8th edition) [18]. The Systemic Immune-Inflammation Index (SII) was calculated using platelet (P), neutrophil (Ne), and lymphocyte (L) counts, according to a previously published formula: SII = P× Ne/L. The neutrophil-to-lymphocyte ratio (NLR) was determined using the formula: NLR = Ne/L [22].

### Treatment outcomes and evaluation

Treatment response and toxicity were assessed according to WHO/ECOG criteria [23]. Survival time was calculated from the initiation of chemotherapy and/or intention-to-treat. Chemotherapy dose reduction was defined as delivery of less than 100% of the planned dose intensity due to treatment modification, including reduction in the number of administered treatment days or use of modified or cytoreductive regimens (e.g., carboplatin-based therapy). Dose reduction did not include inability to complete planned cycles due to early death. Specifically, EP (etoposide and cisplatin) was not classified as dose reduction when used as a therapeutically equivalent alternative to BEP in lower-stage disease.

### Database

We created an internationally shared database to collect de-identified cases of GCT patients that experienced ED defined as death within 3 months from the last cycle of first-line chemotherapy. The database contained data related to patient/tumor characteristics, treatments, and outcome from participating centers across all participating institutions. To assess the relative frequency of ED, the total number of consecutive GCT patients from each participating center were included.

### Statistical analysis

Collected data were tabulated using contingency tables and evaluated for descriptive statistics (mean. standard deviation. median. etc.). The sample size was not based on a prospective power calculation. All consecutive eligible patients from participating centres worldwide were included to maximise statistical power in this rare and clinically challenging population. The relative risk with a 95% confidence interval (CI) was calculated using a 2×2 contingency table. The median follow-up period was calculated as the median observation time among all patients and among those still alive at the time of their last follow-up. Progression-free survival (PFS) was calculated from day 1 of first-line therapy to the date of progression or death while overall survival (OS) was calculated from day 1 of first-line therapy to the date of death. PFS and OS were estimated using the Kaplan-Meier product-limit method and compared between groups by the log-rank test. Univariate analyses with Chi-squared or Fisher’s exact tests were performed for categorical variables. and T-tests or Mann–Whitney U tests were used for continuous variables based on data normality. For all statistical analyses a *p*-value of < 0.05 was considered significant.

## Results

### Patients’ characteristics

We identified 102 patients who experienced ED. The median age was 36 years (range, 18–67) with a median age of 33 years (range, 18–64) among patients with non-seminomatous tumors and 52 years (range, 29–67) among those with pure seminoma. Most (69.6%) of them presented with non-seminomatous GCT, typically originating in the testis (83.3%), and a high proportion (67.6%) had poor-risk disease by IGCCCG criteria. Metastases frequently involved retroperitoneal lymph nodes (73.5%) and lungs (60.8%) while tumor marker elevations were common—particularly LDH (88.2% above the upper limit of normal). Although nearly two-thirds had an initial ECOG performance status of 0 or 1. Baseline tumor markers demonstrated wide variability with median AFP of 37.3 ng/mL (range, 0–435358.0), β-hCG of 1112.5 mIU/mL (range, 0–23,000,000 mIU/mL) and LDH of 10.0 μkat/L (range, 0.1–184.9). The median NLR was 5.6 (range, 0.5–26) and the median SII was 1 419 (range, 23.7–1 2798.6). Patients had a median baseline BMI of 23.3 (range, 17.6–37.2) (Table 1, Supplementary Table 1).

**Table 1:** Patients’ characteristics

Variable	*N*	%
**All patients**	102	100.0
**Histology**		
Pure seminoma	20	19.6
Non-seminoma	71	69.6
No histology	11	10.8
**Histology subtype**		
Embryonal carcinoma	24	23.5
Yolk sac tumor	27	26.5
Choriocarcinoma	20	19.6
Teratoma	18	17.6
Seminoma	30	29.4
Mixed germ cell tumor	37	36.3
Somatic transformation	2	2.0
**Tumor primary**		
Testis	85	83.3
Retroperitoneum	6	5.9
Mediastinum	10	9.8
Unknown	1	1.0
**IGCCCG risk group**		
Good	18	17.6
Intermediate	15	14.7
Poor	69	67.6
**Metastases**		
Retroperitoneal lymph nodes	75	73.5
Lung	62	60.8
Lung involvement > 50%	33	32.4
Liver	41	40.2
Brain	18	17.6
Bone	14	13.7
Supraclavicular lymph nodes	14	13.7
Other	38	37.3
**AFP** > **UNL**	62	60.8
S0	45	44.1
S1	24	23.5
S2	14	13.7
S3	18	17.6
unknown	1	1.0
**beta-HCG** > **UNL**	79	77.5
S0	23	22.5
S1	33	32.4
S2	12	11.8
S3	33	32.4
unknown	1	1.0
**LDH** > **UNL**	90	88.2
S0	9	8.8
S1	5	4.9
S2	75	73.5
S3	10	9.8
**S-stage**		
S0	2	2.0
S1	7	6.9
S2	38	37.3
S3	55	53.9
**Clinical status**		
**ECOG PS**		
0	21	20.6
1	48	47.1
2	17	16.7
3	8	7.8
Unknown	8	7.8
**Resting dyspnea**		
Present	38	37.3
**Hemoptysis**		
Present	12	11.8
**Pain that needs opioids**		
Present	31	30.4
**Comorbidity**		
Present	36	35.3
**Body mass index**		
Less than 18.5	1	1.0
Healthy Weight (18.5 to less than 25)	41	40.2
Overweight (25 to less than 30)	23	22.6
Obesity (30 or greater)	3	2.9
Unknown	34	33.3
**Therapy**		
**Years of therapy**		
2000–2012	41	40.2
2013–2024	60	58.8
**G-CSF support**		
present	47	46.1
**ventilation support**		
present	57	55.9
**ICU transfer**		
present	55	53.9
**Outcome**		
Death within 30 days of therapy	52	51.0
Death within 100 days of therapy	75	73.5
Death between 30 and 100 days	23	22.5
Days more than 100 days	27	26.5

Values shown in bold denote statistically significant differences, defined as *p * < 0.05.

*AFP* Alpha-fetoprotein, *beta-HCG* beta-Human Chorionic Gonadotropin, *ECOG PS* Eastern Cooperative Oncology Group Performance Status, *G-CSF* Granulocyte Colony-Stimulating Factor, *ICU* Intensive Care Unit, *LDH* Lactate Dehydrogenase, *UNL* upper normal limit.

### Treatment patterns

The most administered first-line regimen was BEP (bleomycin, etoposide and cisplatin), received by 58 patients. Other regimens included EP (etoposide and cisplatin) in 32 patients, T-BEP (paclitaxel, bleomycin, etoposide and cisplatin) in 5 patients and carboplatin/EP in 2 patients. Four patients received alternative or non-standard regimens (one patient received cisplatin monotherapy, one patient BEP with carboplatin, one patient CBOP-BEP, and one patient received VAI as part of a CEVAIE-based regimen for a rhabdomyosarcoma component representing somatic differentiation). Median number of administered cycles was 1 (range 0-5). Primary G-CSF was administered to 46.1% of patients; mechanical ventilation and ICU transfer were required in 55.9% and 53.9% of patients, respectively.

Initial chemotherapy dose was reduced in 36.3% of patients, reflecting planned treatment modification at therapy initiation. Dose adaptation decisions were investigator- and site-driven, reflecting local clinical practice patterns and individual patient assessment. Patients with extensive lung involvement ( > 50%) had more often reduced initial dose of chemotherapy compared to patients with less advanced lung disease (57.6% vs. 26.1%, *p* = 0.008). Similarly, patients with very extensive disease ( ≥ 4 more metastatic sites and/or > 50% lung involvement) had more often initial dose reduction compared to patients with less advanced disease (61.4% vs. 19.2%, *p* = 0.00005). However, ECOG ≥ 2 was not associated with significant reduction of initial dose of therapy (*p* = 0.34).

### Causes of deaths

The proportion of patients with ED was 1.67% (range: 0.4–2.1%) of all patients treated with first line chemotherapy. The most common causes of death were acute respiratory failure (ARF) (34.1%), disease progression (16.7%), febrile neutropenia (FN) with septic shock (15.7%), fatal extrapulmonary haemorrhage (12.7%), pulmonary embolism and MI, each with an incidence of 2.0%. Approximately 10.8% of patients died from other causes and in 5.9% of cases the cause of death remained unknown (Table 2). The distribution of causes of death did not differ significantly between patients with pure seminoma and non-seminoma (*p* = 0.29). Acute respiratory failure was the most frequent cause of death in both groups (30.0% vs. 35.4%). Fatal haemorrhage outside the lung (5.0% vs. 14.6%) and death due to disease progression (10.0% vs. 18.3%) occurred more often in non-seminoma patients, whereas a higher proportion of deaths classified as other or unknown causes was observed among seminoma patients (35.0% vs. 12.2%).

**Table 2:** Causes of death

Cause of death	*N*	%	Median (days)	Range (days)
Acute respiratory failure	35	34.3	34	2–150
Disease progression	17	16.7	132	5–167
Septic shock with neutropenia	16	15.7	15	9–170
Fatal haemorrhage in other organ than lung	13	12.8	13	2–113
Other	11	10.8	30	2–179
Unknown	6	5.9	21	6–131
Pulmonary embolism	2	2.0	4	4–11
Myocardial infarction	2	2.0	7	7–17
**All patients**	**102**	**100.0**	**28**	**2–179**

Values shown in bold denote statistically significant differences, defined as *p * < 0.05.

The histogram (Suppl. Fig. 2) illustrates the time distribution of deaths from the initiation of treatment. The highest concentration of deaths occurred within the first 10 days, followed by a continued, though lower, frequency between days 10 and 20. Together, these early events accounted for a substantial proportion of overall mortality, resulting in nearly 50% of all deaths occurring within the first 30 days from treatment initiation. Subsequent intervals showed decreasing frequencies, with approximately 20% of deaths occurring between 80-120 days, and smaller proportions beyond this period.

**Fig. 1:**
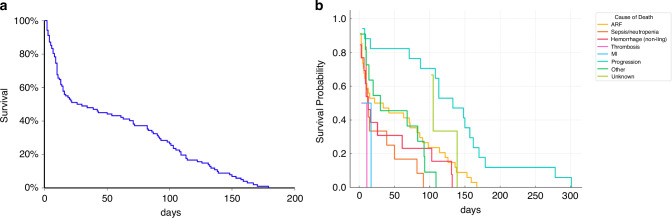
Survival of patients with early death according to time from treatment initiation and cause of death. Kaplan-Meier estimated curve of overall survival (**a**) and according to cause of death (**b**).

Median time from starting of platinum-based therapy to death was 28 days (range, 2–179 days) (Fig. 1a). Patients with disease progression exhibited the longest median survival (132 days, range, 5–167) opposite to deaths due to acute complications such as ARF, septic shock with neutropenia, and/or fatal haemorrhage in non-pulmonary organs occurred earlier with median survival times of 34, 15, and 13 days, respectively. The shortest survival was observed in patients who died from pulmonary embolism and MI, 4 and 7 days, respectively. (Table 2, Fig. 1b). Median overall survival (OS) differed significantly by chemotherapy regimen (*p* = 0.013): BEP, 71 days (95% CI 21–91); EP, 17 days (95% CI 6–34); and other regimens, 10 days (95% CI 2–39). Dose reduction was associated with inferior overall survival with a median OS of 11 days (95% CI 9–16) in patients with dose reduction compared with 61 days (95% CI 21–90) in those without dose reduction (*p* = 0.045).

### Factors associated with development of acute respiratory failure

Patients with ARF were older with a median age of 43 years compared to 33 years in the no-ARF group (*p* = 0.06). Among patients with ARF, significantly more had lung involvement greater than 50% (51.4% vs. 22.4%, *p* = 0.004), resting dyspnoea (57.1% vs. 26.9%. *p* = 0.005) and haemoptysis (22.9% vs. 6.0%. *p* = 0.021) compared with those without ARF. Pretreatment β-hCG (median, mIU/mL 5893.1 vs. 1060.0; *p* = 0.18) and LDH (median, µkat/L 148.0 vs. 722.0; *p* = 0.07) levels were not significantly higher in patients who experienced ARF compared with those who did not develop ARF. In contrast, pretreatment AFP levels were significantly lower in patients who developed ARF (median, ng/mL 5.8 vs. 7.0; *p* = 0.02). Other factors, including histology and comorbidities, and or reduction of initial dose of chemotherapy did not differ significantly between the two groups (Supplementary Tables 2, 3).

### Factors associated with death within 30 days from starting chemotherapy

Patients who died within 30 days, exhibited a notably higher disease burden and poorer clinical status. They were also more likely to have no histology reported, reflecting urgent clinical situation, where was no time to obtain histology. Moreover, these patients had more frequently retroperitoneal lymph node and liver metastases, and extensive lung involvement. In terms of tumor markers, a higher proportion had β-HCG S3 and overall S3 staging. Clinically, these patients presented with numerically worse ECOG performance status (ECOG ≥ 2, 28.9% vs. 20.0%, *p* = 0.36) and more frequently required ventilator (67.3% vs. 44.0%) or ICU support (67.3% vs. 40.0%). Very early deaths were primarily attributable to acute complications such as ARF or haemorrhage, in contrast to later deaths, which were more often related to disease progression (Table 3). While age and BMI did not differ significantly between groups, patients in the very early-mortality group had markedly elevated inflammatory markers, with higher median NLR and SII index (Supplementary Table 4).

**Table 3:** Comparison of patients/disease characteristics between patients that died within 30 days of start of therapy comparison to more than 30 days (categorical variables)

Variable	more than 30 days		within 30 days		
	***N***	**%**	***N***	**%**	***p***-**value**
**All patients**	50	100.0	52	100.0	NA
**Cause of death**					
Acute respiratory failure	18	36.0	17	32.7	**0.044**
Septic shock with neutropenia	6	12.0	10	19.2	
Fatal hemorrhage in other organ than lungs lung	4	8.0	9	17.3	
Pulmonary embolism	0	0.0	2	4.0	
Myocardial infarction	0	0.0	2	4.0	
Disease progression	14	26.9	3	6.0	
Other	5	9.6	6	12.0	
Unknown	3	5.8	3	6.0	
**Histology**					
Pure seminoma	10	20.0	10	19.2	1.000
Non-seminoma	38	76.0	33	63.5	0.200
No histology	2	4.0	9	17.3	0.052
**Histology subtype**					
Embryonal carcinoma	17	34.0	7	13.5	**0.018**
Yolk sac tumor	16	32.0	11	21.2	0.256
Choriocarcinoma	8	16.0	12	23.1	0.453
Teratoma	10	20.0	8	15.4	0.605
Seminoma	16	32.0	14	26.9	0.661
Mixed germ cell tumor	20	40.0	17	32.7	0.673
Somatic transformation	2	4.0	0	0.0	0.495
**Tumor primary**					
Testis	38	76.0	47	90.4	0.094
Retroperitoneum	3	6.0	3	5.8	
Mediastinum	8	16.0	2	3.8	
Unknown	1	2.0	0	0.0	
**IGCCCG risk group**					
Good	8	16.0	10	19.2	0.325
Intermediate	10	20.0	5	9.6	
Poor	32	64.0	37	71.2	
**Metastases**					
Retroperitoneal lymph nodes	30	60.0	45	86.5	**0.023**
Lung	26	52.0	36	69.2	0.207
Lung involvement > 50%	8	16.0	25	48.1	**0.001**
Liver	14	28.0	27	51.9	0.061
Brain	10	20.0	8	15.4	0.438
Bone	8	16.0	6	11.5	0.401
Supraclavicular lymph nodes	8	16.0	6	11.5	0.401
Other	13	26.0	25	48.1	0.058
**AFP**					
S0	18	36.0	27	51.9	0.394
S1	14	28.0	10	19.2	
S2	8	16.0	6	11.5	
S3	10	20.0	8	15.4	
unknown	0	0.0	1	1.9	
**beta-HCG**					
S0	14	28.0	9	17.3	**0.019**
S1	20	40.0	13	25.0	
S2	7	14.0	5	9.6	
S3	9	18.0	24	46.2	
unknown	0	0.0	1	1.9	
**LDH**					
S0	4	8.0	5	9.6	0.792
S1	2	4.0	3	5.8	
S2	40	80.0	35	67.3	
S3	4	8.0	6	11.5	
unknown	0	0.0	2	3.8	
**S-stage**					
S0	2	4.0	0	0.0	**0.012**
S1	2	4.0	5	9.6	
S2	25	50.0	13	25.0	
S3	21	42.0	34	65.4	
unknown					
**Clinical status**					
**ECOG PS**					
0	8	16.0	13	25.0	**0.005**
1	26	52.0	22	42.3	
2	10	20.0	7	13.5	
3	0	0.0	8	15.4	
unknown	6	12.0	2	3.8	
**Resting dyspnea**	17	34.0	21	40.4	0.544
**Hemoptysis**	5	10.0	7	13.5	0.761
**Pain that needs opioids**	14	28.0	17	32.7	0.670
**Comorbidity**	17	34.0	19	36.5	0.836
**Body mass index**					
Less than 18.5	1	2.0	0	0.0	0.059
Healthy Weight (18.5 to less than 25)	17	34.0	24	46.2	
Overweight (25 to less than 30)	8	16.0	15	28.8	
Obesity (30 or greater)	3	6.0	0	0.0	
**Therapy**					
**Years of therapy**					
2000–2012	20	40.0	21	40.4	1.000
2013–2024	29	58.0	31	59.6	
**G-CSF support**	24	48.0	23	44.2	0.843
**Ventilation support**	22	44.0	35	67.3	**0.028**
**ICU transfer**	20	40.0	35	67.3	**0.009**

Values shown in bold denote statistically significant differences, defined as *p * < 0.05.

*AFP* Alpha-fetoprotein, *beta-HCG* beta-Human Chorionic Gonadotropin, *ECOG PS* Eastern Cooperative Oncology Group Performance Status, *G-CSF* Granulocyte Colony-Stimulating Factor, *ICU* Intensive Care Unit *LDH* Lactate Dehydrogenase, *UNL* upper normal limit.

## Discussion

This international study sheds light on the rarely reported events of ED among GCT patients. Compared with the IGCCCG Update populations, our cohort represents a highly selected subgroup with exceptionally adverse disease characteristics. When stratified by histology, patients with non-seminomatous tumors were slightly older than those in the IGCCCG non-seminoma cohort (median 33 vs. 29 years), whereas patients with pure seminoma were substantially older (median 52 vs. 38 years) [3, 4]. Consistent with the concept of “super-high-risk” GCTs —defined as IGCCCG poor-risk disease with additional adverse clinical features such as older age, liver metastases, extensive lung involvement, and markedly elevated tumor markers—most patients in our cohort fulfilled these criteria, with pronounced pulmonary involvement, frequent liver metastases, and predominantly S2–S3 tumor marker stages. Compared with the general GCT population, these patients represent an extreme and particularly vulnerable subset not fully captured by standard IGCCCG risk stratification [3, 4].

Our data showed that roughly half of the patients died within 30 days of starting chemotherapy, a period often overlooked in both trials and routine audits. This left-skewed distribution highlights a challenging early prognosis for a substantial portion of the patient cohort. These ultra-early deaths tend to be driven by acute toxicities, respiratory failure, haemorrhage, and infection rather than tumor progression per se. Conversely, deaths occurring beyond the 30-day mark were more frequently related to disease progression, likely reflecting resistant tumor biology or cumulative organ dysfunction. Importantly, the timing of death appears to differentiate preventable from less-modifiable causes. Events within the first 10–14 days, particularly due to FN, sepsis, or ARF, may be amenable to rapid recognition, early ICU involvement, and anticipatory supportive measures [5, 8]. The shortest survival was observed in patients who died from pulmonary embolism and MI, 4 and 7 days, respectively, underscoring the sudden and severe nature of these events.

Acute respiratory failure emerged as the most common cause of ED. Traditionally linked to choriocarcinoma syndrome, ARF in our cohort was not significantly associated with any histology subtype including choriocarcinoma, while numerically, higher number of patients had no histology before starting of therapy, highlighting the aggressive behaviour of the disease, with urgent need of therapy even before obtaining the histology. This risk was associated with dyspnoea at baseline, haemoptysis, poor performance status, massive pulmonary involvement ( > 50% of lung parenchyma), but not with β-HCG level. This finding challenges conventional definitions and suggests that the term “choriocarcinoma syndrome” may inadequately capture the broader spectrum of respiratory complications in GCTs. From a patho-physiological perspective, ARF in these patients may result from a combination of tumor necrosis, haemorrhage into tumor-laden lungs, chemotherapy-induced endothelial damage, and secondary infections in the setting of neutropenia [10]. Interestingly, despite theoretical concerns, classic tumor lysis syndrome was not observed, with no metabolic derangements supporting its presence. These observations point to a distinct mechanism of respiratory compromise that warrants further investigation [10].

Febrile neutropenia and extrapulmonary haemorrhage each accounted for a significant proportion of Eds, particularly in the first two weeks post-treatment initiation. Retrospective analyses of microbiological data revealed that empirical antibiotic coverage was often suboptimal, and all autopsied patients had evidence of unrecognized or inadequately treated infections [7]. This highlights a critical gap in the pre-emptive management of infectious complications. Early implementation of infection control measure, thoracic imaging, early bronchoscopy, blood cultures, and empiric antibiotics active against Gram-negative and resistant Gram-positive organisms should become standard care for super-high-risk patients [7]. Importantly, only half of the patients in our study were admitted to ICU, received mechanical ventilation or G-CSF, despite presenting with complications warranting intensive support. This discrepancy reflects either late recognition of deterioration or institutional differences in ICU admission thresholds. Single institution experience supports early admission to ICU as effective approach to decrease mortality in this patient’s population [5].

Though less common, fatal cardiovascular events such as pulmonary embolism (PE) and MI were observed. These findings underscore the hypercoagulable state associated with high tumor burden, elevated LDH, and pro-inflammatory cytokine release [24–27]. Despite mounting concern over thrombotic risks in GCTs, current anticoagulation trials largely exclude super-high-risk patients due to bleeding concerns, leaving clinicians without evidence-based guidelines for thromboprophylaxis in this setting [28–30]. The need for individualized risk-benefit assessment remains paramount.

The association between dose reduction and inferior overall survival observed in our cohort is likely influenced by indication bias, as treatment attenuation was preferentially applied in clinically frailer patients with poorer baseline status. Similarly, full-dose BEP was more commonly administered to clinically fitter patients, whereas alternative regimens were considered in those with advanced disease, organ dysfunction, or limited tolerance to standard cisplatin-based therapy, suggesting that observed survival differences may primarily reflect patient selection rather than a direct treatment effect.

A substantial proportion of patients (44.3%) received reduced-intensity chemotherapy at initiation, primarily based on organ dysfunction or anticipated pulmonary complications. This clinical decision-making reflects the challenging balance between maintaining curative intent and mitigating early life-threatening toxicity in super-high-risk GCT patients. In several cases, patients who received attenuated doses still succumbed to respiratory failure or other complications. The rationale behind dose reduction is to avoid overwhelming tumor lysis and inflammatory response, yet underdosing may leave bulky tumor masses partially intact, perpetuating local complications such as haemorrhage, necrosis, and infection [5, 16]. The lack of randomized data comparing dose-intensified versus dose-reduced regimens in super-high-risk GCTs remains a major unmet need. Regimens such as Baby-BOP or stepwise cisplatin escalation are promising, but their validation in multicentre prospective trials is urgently required [5, 16, 19, 20].

Despite most patients being treated in tertiary or expert centers, supportive care interventions such as mechanical ventilation, G-CSF were inconsistently applied. This reflects significant heterogeneity in institutional practice and highlights the need for standardized clinical pathways to ensure timely escalation of care. Centralization of care is necessary, but insufficient unless paired with robust early warning systems, triage algorithms, and intensive monitoring protocols for super-high-risk presentations. Decision-making regarding ICU admission, chemotherapy modification, or antimicrobial coverage should be guided not only by institutional capacity but also by validated risk stratification tools – predicting nomograms.

Our findings reinforce the existence of a “super-high-risk” phenotype in GCTs—characterized by very high β-hCG, massive lung involvement, liver metastases, ECOG ≥ 2, and activated systemic inflammation. Patients with this profile face a high risk of ultra-early mortality and should be managed proactively. Based on these observations we suggest: (i) immediate triage to experienced, high-volume centers upon presentation; (ii) early ICU involvement for those with respiratory compromise or rapid clinical deterioration; (iii) tailored chemotherapy induction, potentially using reduced-intensity regimens and delayed bleomycin to mitigate early toxicity; (iv) pre-emptive supportive care, including antimicrobial prophylaxis, G-CSF, and individualized thromboprophylaxis; (v) use of inflammatory biomarkers for early risk stratification and treatment adaptation; and vi) inclusion in registries or observational studies to ensure adequate representation and inform evidence-based care for this vulnerable subgroup.

Even though this is a first multicentric study aimed to characterize this patient’s population, this study has several limitations. The retrospective design, limited sample size, and tertiary center recruitment may introduce referral and survivorship bias. Some relevant variables such as timing of interventions, adherence to protocols, and precise cause of death were inconsistently recorded or classified. Nonetheless, our findings align with previous reports and expand upon them by offering a comprehensive, multicentre perspective. Future prospective efforts should focus on decision-making regarding ICU admission, chemotherapy modification, or antimicrobial coverage should be guided not only by institutional capacity but also by validated risk stratification tools – predicting nomograms.

## Conclusions

Early mortality in metastatic GCTs comprises no more than 2.1% of all cases but it remains a critical concerning issue. Most deaths occur within the first month of chemotherapy and are driven by complications such as ARF, infection, and haemorrhage rather than disease progression. These deaths cluster in a well-defined subset of “super-high-risk” patients whose management requires specialized care, proactive supportive strategies, and possibly modified induction therapy. Recognition, triage, and rapid escalation of care are key to improving outcomes. To address this, remote monitoring and stringent surveillance strategies in those at greatest risk of such complications should be developed and disseminated across national and society-level guidelines. Bridging the knowledge gap through prospective studies and harmonized protocols will be essential to mitigate avoidable deaths in this otherwise highly curable cancer.

### Global society for rare GU tumors

All members of the Global Society for Rare GU Tumors who contributed to this manuscript and qualify for authorship are listed individually in the main author list above; therefore, no additional consortium authors are listed separately.

## Supplementary information

Suppl material CLEANED

## Data Availability

Study data are available upon request
