# Supplementary material for: Risk factors and causes of early death in germ cell tumors: a Global Society for Rare GU tumors study
Source: Br J Cancer. 2026 Jun 2;135(5):728–35. doi: 10.1038/s41416-026-03484-0 (PMC13478367; doi:10.1038/s41416-026-03484-0)
Supplement: Supplementary file 1 [file 41416_2026_3484_MOESM1_ESM.doc]

**Supplementary Table 1. Pretreatment levels of selected factors**

|  | **median** | **min** | **max** |
| --- | --- | --- | --- |
| Age | 36 | 18 | 67 |
| Pretreatment AFP (ng/mL) | 37.3 | 0.0 | 435358.0 |
| Pretreatment beta-HCG (IU/mL) | 1112.5 | 0.0 | 23000000.0 |
| Pretreatment LDH (μkat/L) | 10.0 | 0.1 | 184.9 |
| pretreatment NLR | 5.6 | 0.5 | 26.0 |
| pretreatment SII | 1419.0 | 23.7 | 12798.6 |
| Baseline BMI | 23.3 | 17.6 | 37.2 |

**Abbreviations:** AFP, Alpha-fetoprotein, beta-HCG: beta-Human Chorionic Gonadotropin: BMI, Body Mass Index: LDH, Lactate Dehydrogenase: NLR, Neutrophil-to-Lymphocyte Ratio: SII, Systemic Immune-Inflammation Index

**Supplementary Table 2.** Factors associated with development of acute respiratory failure

| **Variable** | **No-ARF** |  | **ARF** |  |  |
| --- | --- | --- | --- | --- | --- |
|  | **yes** |  | **yes** |  | **p-value** |
|  | **N** | **%** | **N** | **%** |  |
| **All patients** | 67 | 100.0 | 35 | 100.0 | NA |
| **histology** |  |  |  |  |  |
| Pure seminoma | 14 | 20.9 | 6 | 17.1 | 0.795 |
| Non-seminoma | 48 | 71.6 | 23 | 65.7 | 0.651 |
| No histology | 5 | 7.5 | 6 | 17.1 | 0.180 |
| Embryonal carcinoma | 15 | 22.4 | 9 | 25.7 | 0.808 |
| Yolk sac tumor | 19 | 28.4 | 8 | 22.9 | 0.634 |
| Choriocarcinoma | 13 | 19.4 | 7 | 20.0 | 1.000 |
| Teratoma | 14 | 20.9 | 4 | 11.4 | 0.276 |
| Seminoma | 18 | 26.9 | 12 | 34.3 | 0.644 |
| Mixed germ cell tumor | 22 | 32.8 | 15 | 42.9 | 0.375 |
| Somatic transformation | 0 | 0.0 | 2 | 5.7 | 0.116 |
| **Tumor primary** |  |  |  |  |  |
| Testis | 56 | 83.6 | 29 | 82.9 | 0.704 |
| Retroperitoneum | 3 | 4.5 | 3 | 8.6 |  |
| Mediastinum | 7 | 10.4 | 3 | 8.6 |  |
| Unknown | 1 | 1.5 | 0 | 0.0 |  |
| **IGCCCG risk group** |  |  |  |  |  |
| Good | 15 | 22.4 | 3 | 8.6 | 0.187 |
| Intermediate | 9 | 13.4 | 6 | 17.1 |  |
| Poor | 43 | 64.2 | 26 | 74.3 |  |
| **Metastases** |  |  |  |  |  |
| Retroperitoneal lymph nodes | 50 | 74.6 | 25 | 71.4 | 0.289 |
| Lung | 39 | 58.2 | 23 | 65.7 | 0.112 |
| Lung involvement >50% | 23 | 34.3 | 18 | 51.4 | **0.024** |
| Liver | 10 | 14.9 | 8 | 22.9 | 0.167 |
| Brain | 13 | 19.4 | 1 | 2.9 | 0.057 |
| Bone | 10 | 14.9 | 4 | 11.4 | 1.000 |
| Supraclavicular lymph nodes | 25 | 37.3 | 13 | 37.1 | 0.651 |
| Other | 15 | 22.4 | 18 | 51.4 | **0.004** |
| **AFP** |  |  |  |  |  |
| S0 | 23 | 34.3 | 22 | 62.9 | **0.003** |
| S1 | 18 | 26.9 | 6 | 17.1 |  |
| S2 | 9 | 13.4 | 5 | 14.3 |  |
| S3 | 17 | 25.4 | 1 | 2.9 |  |
| unknown | 0 | 0.0 | 1 | 2.9 |  |
| **beta-HCG** |  |  |  |  |  |
| S0 | 17 | 25.4 | 6 | 17.1 | 0.597 |
| S1 | 23 | 34.3 | 10 | 28.6 |  |
| S2 | 8 | 11.9 | 4 | 11.4 |  |
| S3 | 19 | 28.4 | 14 | 40.0 |  |
| unknown | 0 | 0.0 | 1 | 2.9 |  |
| **LDH** |  |  |  |  |  |
| S0 | 6 | 9.0 | 3 | 8.6 | 0.155 |
| S1 | 5 | 7.5 | 0 | 0.0 |  |
| S2 | 49 | 73.1 | 26 | 74.3 |  |
| S3 | 5 | 7.5 | 5 | 14.3 |  |
| unknown | 2 | 3.0 | 1 | 2.9 |  |
| **S-stage** |  |  |  |  |  |
| S0 | 1 | 1.5 | 1 | 2.9 | 0.625 |
| S1 | 6 | 9.0 | 1 | 2.9 |  |
| S2 | 24 | 35.8 | 14 | 40.0 |  |
| S3 | 36 | 53.7 | 19 | 54.3 |  |
| **ECOG PS** |  |  |  |  |  |
| 0 | 17 | 25.4 | 4 | 11.4 | **0.005** |
| 1 | 35 | 52.2 | 13 | 37.1 |  |
| 2 | 12 | 17.9 | 5 | 14.3 |  |
| 3 | 1 | 1.5 | 7 | 20.0 |  |
| unknown | 2 | 3.0 | 6 | 17.1 |  |
| **Resting dyspnoe** | 18 | 26.9 | 20 | 57.1 | **0.005** |
| **Hemoptysis** | 4 | 6.0 | 8 | 22.9 | **0.021** |
| **Pain that needs opioids** | 20 | 29.9 | 11 | 31.4 | 1.000 |
| **Comorbidity** | 22 | 32.8 | 14 | 40.0 | 0.520 |
| **Years of therapy** |  |  |  |  |  |
| 2000-2012 | 30 | 44.8 | 11 | 31.4 | 0.286 |
| 2013-2024 | 37 | 55.2 | 23 | 65.7 |  |
| **G-CSF support** | 34 | 50.7 | 13 | 37.1 | 0.215 |
| **BMI** |  |  |  |  |  |
| Less than 18.5. | 1 | 1.5 | 0 | 0.0 | 0.062 |
| Healthy Weight. 18.5 to less than 25. | 26 | 38.8 | 15 | 42.9 |  |
| Overweight. 25 to less than 30. | 16 | 23.9 | 7 | 20.0 |  |
| Obesity. 30 or greater. | 0 | 0.0 | 3 | 8.6 |  |
| Unknown | 24 | 35.8 | 10 | 28.6 |  |
| **Bleomycin** | 42 | 62.7 | 16 | 45.7 | 0.140 |
| **Dose reduction** | 22 | 32.8 | 15 | 42.9 | 0.174 |

**Abbreviations:** AFP, Alpha-fetoprotein, ARF, acute respiratory failure, beta-HCG: beta-Human Chorionic Gonadotropin, ECOG PS, Eastern Cooperative Oncology Group Performance Status, G-CSF, Granulocyte Colony-Stimulating Factor, ICU, Intensive Care Unit, LDH, Lactate Dehydrogenase, UNL, upper normal limit

**Supplementary Table 3.** Comparison of patients/disease characteristics between patients that developed ARF compared to patients with no-ARF (continuous variables).

| **Variable** | **N** | **mean** | **median** | **SD** | SEM | **p-value** |
| --- | --- | --- | --- | --- | --- | --- |
| **Age** |  |  |  |  |  |  |
| ARF | 35 | 41.5 | 43.0 | 13.2 | 2.3 | 0.061 |
| no-ARF | 67 | 36.2 | 33.0 | 13.5 | 1.6 |  |
| **NLR** |  |  |  |  |  |  |
| ARF | 28 | 8.0 | 6.9 | 5.6 | 1.1 | 0.122 |
| no-ARF | 66 | 6.5 | 4.8 | 6.0 | 0.7 |  |
| **SII** |  |  |  |  |  |  |
| ARF | 28 | 2589.2 | 1916.1 | 2509.9 | 479.1 | 0.263 |
| no-ARF | 66 | 2222.9 | 1198.4 | 2545.8 | 312.1 |  |
| **Body mass index** |  |  |  |  |  |  |
| ARF | 22 | 24.1 | 23.0 | 3.8 | 1.0 | 0.953 |
| no-ARF | 46 | 24.4 | 23.9 | 4.8 | 0.7 |  |

**Abbreviations:** ARF, acute respiratory failure, NLR, Neutrophil-to-Lymphocyte Ratio, SII, Systemic Immune-Inflammation Index

**Supplementary Table 4.** Comparison of patients/disease characteristics between patients that died within 30 days of start of therapy comparison to more than 30 days (continuous variables).

| **Variable** | **N** | **mean** | **median** | **SD** | **SEM** | **p-value** |
| --- | --- | --- | --- | --- | --- | --- |
| **Age (years)** |  |  |  |  |  |  |
| more than 30 days | 50 | 38.4 | 35.5 | 13.5 | 1.9 | 0.730 |
| within 30 days | 52 | 37.7 | 36.5 | 13.7 | 1.9 |  |
| **NLR** |  |  |  |  |  |  |
| more than 30 days | 44 | 5.2 | 3.0 | 4.9 | 0.9 | **0.007** |
| within 30 days | 50 | 8.6 | 7.4 | 6.3 | 0.8 |  |
| **SII** |  |  |  |  |  |  |
| more than 30 days | 44 | 1659.2 | 746.2 | 2111.4 | 370.8 | **0.003** |
| within 30 days | 50 | 2924.1 | 2160.8 | 2728.7 | 347.8 |  |
| **Body mass index** |  |  |  |  |  |  |
| more than 30 days | 26 | 24.2 | 23.0 | 4.5 | 0.9 | 0.791 |
| within 30 days | 42 | 24.4 | 23.7 | 4.6 | 0.7 |  |

**Abbreviations:** NLR, Neutrophil-to-Lymphocyte Ratio, SII, Systemic Immune-Inflammation Index

**Supplementary Figure 1.** Study Flow diagram

Assessed for eligibility

(n = 109)

Excluded (n = 7)

- > 1 line of therapy (n=6)
- Death > 3 months after finishing 1^st^ line therapy (n=1)

Analyzed

(n = 102)

Death within 30 days of therapy (n = 52)

Death > 30 days after the therapy (n = 50)

Acute respiratory failure (n = 17)

Other causes of death (n = 35)

Acute respiratory failure (n = 18)

Other causes of death (n = 32)

**Supplementary Figure 2.** Distribution of early death from treatment initiation

days
